# Taxonomic evaluation of *Xylodon* (Hymenochaetales, Basidiomycota) in Korea and sequence verification of the corresponding species in GenBank

**DOI:** 10.7717/peerj.12625

**Published:** 2021-12-10

**Authors:** Yoonhee Cho, Ji Seon Kim, Yu-Cheng Dai, Yusufjon Gafforov, Young Woon Lim

**Affiliations:** 1 School of Biological Sciences and Institute of Microbiology, Seoul National University, Seoul, South Korea; 2 Institute of Microbiology, School of Ecology and Nature Conservation, Beijing Forestry University, Beijing, China; 3 Laboratory of Mycology, Institute of Botany, Academy of Sciences of Republic of Uzbekistan, Tashkent, Uzbekistan

**Keywords:** Hyphodontia, ITS, nrLSU, Phylogeny, Schizoporaceae, Schizopora, White-rot, Wood decay fungus

## Abstract

Genus *Xylodon* consists of white-rot fungi that grow on both angiosperms and gymnosperms. With resupinate and adnate basidiomes, *Xylodon* species have been classified into other resupinate genera for a long time. Upon the integration of molecular assessments, the taxonomy of the genus has been revised multiple times over the years. However, the emendations were poorly reflected in studies and public sequence databases. In the present study, the genus *Xylodon* in Korea was evaluated using molecular and morphological analyses of 172 specimens collected in the period of 2011 to 2018. The host types and geographical distributions were also determined for species delimitation. Furthermore, public sequences that correspond to the *Xylodon* species in Korea were assessed to validate their identities. Nine *Xylodon* species were identified in Korea, with three species new to the country. Morphological differentiation and identification of some species were challenging, but all nine species were clearly divided into well-resolved clades in the phylogenetic analyses. Detailed species descriptions, phylogeny, and a key to *Xylodon* species in Korea are provided in the present study. A total of 646 public ITS and nrLSU sequences corresponding to the nine *Xylodon* species were found, each with 404 (73.1%) and 57 (61.3%) misidentified or labeled with synonymous names. In many cases, sequences released before the report of new names have not been revised or updated. Revisions of these sequences are arranged in the present study. These amendments may be used to avoid the misidentification of future sequence-based identifications and concurrently prevent the accumulation of misidentified sequences in GenBank.

## Introduction

The genus *Xylodon* (Pers.) Fr. is the oldest genus in Schizoporaceae Jülich of the Hymenochaetales. It is characterized by resupinate basidiomes with various hymenophore types, including grandinioid, odontioid, and poroid hymenophores, as well as monomitic to trimitic hyphal systems, different types of cystidia including capitate, clavate, moniliform, and subulate cystidia, basidia with four sterigmata and basal clamp connections, and narrow to wide subglobose or ellipsoid basidiospores that are inamyloid and colorless (Hjortstam & Ryvarden, 2009; Riebesehl & Langer, 2017). Many of these morphological characteristics are shared with other genera in Schizoporaceae. For a long time, odontioid resupinate species have been placed in *Hyphodontia*. However, phylogenetic studies have shown that many misaligned species with odontioid and poroid hymenophores belong to the genus *Xylodon* (Larsson et al., 2006; Hjortstam & Ryvarden, 2009; Yurchenko & Wu, 2016). Currently, *Xylodon* is the largest genus within the family Schizoporaceae and encompasses species within the genera *Lagarobasidium* (Viner et al., 2018), *Odontiopsis*, and *Palifer* (Riebesehl et al., 2019) as synonyms. The genus *Schizopora* has also been integrated into *Xylodon* because of its synonymous morphological characteristics and indifferentiable sequences (Riebesehl & Langer, 2017).

*Xylodon* species inhabit a wide range of niches worldwide, including temperate, tropical, or subtropical regions of America (Langer, 1994), Asia (Langer, 1994; Chen, Wu & Chen, 2018), and Europe (Eriksson & Ryvarden, 1976; Riebesehl et al., 2019). They grow on various angiosperm and gymnosperm species, as well as ferns such as *Cyathea* (Riebesehl et al., 2019). They play an important role in the ecosystem as white-rot fungi, degrading some of the most recalcitrant macromolecules with extracellular ligninolytic enzymes (Nguyen et al., 2021). These enzymes may be practically used in biotechnology. For example, *X. paradoxus* has been shown to have high oxidative enzyme activities (Volobuev, 2020), and *X*. *flaviporus* has potential bioremediation abilities to degrade organic pollutants such as polycyclic aromatic hydrocarbons (Lee et al., 2014). In addition to their enzymatic abilities, the compounds of *X. flaviporus* also have medicinal effects, inhibiting RANKL-stimulated osteoclastogenesis (Kwon et al., 2019).

Taxonomic studies on many *Xylodon* species in Korea were primarily based on morphology and less on genetic assessment (Lim & Jung, 2001; Lee & Jung, 2005). In addition, the transition of old names to the genus *Xylodon* has not been reflected in some species in Korea to date. The later integration of phylogenetic analyses has greatly increased the accuracy of the classification and identification of *Xylodon* species. However, public databases contain numerous reference sequences that differ in species identities, making it difficult to accurately identify query sequences with certainty (Nilsson et al., 2006; Jung et al., 2014; Jargalmaa et al., 2017). In the present study, we reflected the recent changes made in the classification and taxonomy of *Xylodon* in Korea and evaluated the public database to validate the sequence identities and rectify misidentified sequences. This study serves as a standard to accurately differentiate and identify *Xylodon* species in Korea, regardless of the methods (either molecular or morphological approach).

## Materials & Methods

### Specimen collection

A total of 172 specimens (Table 1) were collected in the period of 2011 to 2018 in South Korea. They were obtained from institutions for fungal collection in Korea, namely Korea National Arboretum (KA), National Institute of Biological Resources of Korea (NIBR), and Seoul National University Fungus Collection (SFC) as dried specimens. The host type of each species was analyzed based on the specimen collection information (Table 1).

**Table 1:** *Xylodon* specimens collected from Korea and their host type.

Identity	Strains		
	Angiosperms	Gymnosperms	No description
*Xylodon asperus*	–	SFC20170209-01, SFC20170209-11, SFC20170209-13, SFC20180410-30, SFC20180426-01, SFC20110519-18	SFC20121130-02
*X. flaviporus*	SFC20120820-08, SFC20140313-22, SFC20140529-03, SFC20140529-14, SFC20140530-01, SFC20140530-04, SFC20140921-05, SFC20140926-17, SFC20150320-07, SFC20150404-03, SFC20150404-07, SFC20150514-06, SFC20150526-04, SFC20150625-24, SFC20150626-06, SFC20150707-62, SFC20150715-09, SFC20150716-10, SFC20150909-13, SFC20160114-06, SFC20160602-19, SFC20160614-40, SFC20160621-05, SFC20160629-02, SFC20160811-04, SFC20160812-38, SFC20160816-01, SFC20160906-02, SFC20160920-08, SFC20160922-02, SFC20160922-08, SFC20170209-03, SFC20170430-09, SFC20170524-07, SFC20170807-04, SFC20170808-21, SFC20170831-06, SFC20170908-67, SFC20180410-17, SFC20180704-47, SFC20180705-20	SFC20120926-26, SFC20150407-05, SFC20150902-27, SFC20150908-38, SFC20160909-12, SFC20180710-24	SFC20110921-10, SFC20110921-35, SFC20111001-50, SFC20111001-84, SFC20120409-11, SFC20120410-10, SFC20120508-01, SFC20120601-03, SFC20120601-11, SFC20120919-65, SFC20130315-25, SFC20130404-05, SFC20130521-43, SFC20130521-49, SFC20130719-41, SFC20130917-15, SFC20140412-07, SFC20140530-03, SFC20150129-03, SFC20150501-03, SFC20150701-12, SFC20160114-27, SFC20160114-32, SFC20160126-18, SFC20160127-07, SFC20160527-52, SFC20160726-31, SFC20170705-08, SFC20170920-29, SFC20180410-29, SFC20180524-06, SFC20180705-85, SFC20180802-03
*X. kunmingensis*	SFC20160114-24, SFC20170317-07	–	–
*X. nespori*	–	SFC20120601-18, SFC20150523-08	–
*X. niemelaei*	KUC20160721B-26		
*X. ovisporus*	SFC20110823-19, SFC20120410-26, SFC20120726-01, SFC20121009-17, SFC20121009-34, SFC20130403-08, SFC20130521-61, SFC20130730-29, SFC20140410-02, SFC20140411-02, SFC20140911-31, SFC20140926-20, SFC20150516-05, SFC20150518-12, SFC20150527-11, SFC20150625-33, SFC20150707-80, SFC20150716-02, SFC20160114-21, SFC20160225-08, SFC20160225-14, SFC20160526-13, SFC20160527-02, SFC20160712-07, SFC20160811-11, SFC20160817-23, SFC20160908-39, SFC20170208-11, SFC20170221-03, SFC20170221-09, SFC20170228-01, SFC20170317-10, SFC20170430-11, SFC20170713-32, SFC20180207-01, SFC20180523-10, SFC20180720-01, SFC20180807-06, SFC20180810-02	SFC20150407-06, SFC20160512-31, SFC20160811-36, SFC20160920-29, SFC20170718-08, SFC20171018-07	SFC20110823-19, SFC20120410-26, SFC20120726-01, SFC20121009-17, SFC20130403-08, SFC20130521-61, SFC20130730-29, SFC20140410-02, SFC20140411-02, SFC20140926-20, SFC20150516-05, SFC20160114-21, SFC20170430-11, SFC20170713-32, SFC20180207-01, SFC20180523-10, SFC20180720-01, SFC20180807-06, SFC20180810-02
*X. serpentiformis*	KUC20121019-31	–	–
*X. spathulatus*	–	SFC20180710-20, SFC20180818-36	–
*X. subflaviporus*	SFC20120821-53, SFC20150514-14, SFC20150522-08, SFC20160628-20, SFC20160708-32, SFC20161012-15, SFC20180818-15	SFC20170316-24, SFC20170316-25, SFC20170426-14	SFC20150701-67, SFC20150707-63, SFC20180808-08

### Morphological observations

All specimens were preliminarily grouped with respect to their macromorphological characteristics, including hymenophore types, aculei length, and pore size. Subsequently, micromorphological features, including cystidia types and basidiospore size, were observed using one to six well-preserved specimens of each group. Pieces of dried specimens were mounted in 5% KOH. Observations were performed under a Nikon 80i compound light microscope (Nikon, Tokyo, Japan) at 400× to 1,000× magnification. For each specimen, 20 basidia and 30 basidiospores were measured when possible. Basidiospore dimensions were expressed as the range of minimum to maximum length and width. “Q” refers to the average length to width ratio of basidiospores.

### DNA extraction, PCR, and sequencing

Small hymenophore pieces obtained from 1 to 10 representative specimens of each group were peeled off from the wood using sterile forceps, and each was placed in 200 μL of 2× CTAB buffer. Besides the representative specimens, other ambiguous specimens, including juvenile specimens or those too bad in state, were also analyzed. Genomic DNA extraction was conducted using the AccuPrep Genomic DNA Extraction Kit (Bioneer, Daejeon, Korea) according to the manufacturer’s protocol with a modification where sterile micropestles were used to grind the tissue samples. PCR was performed using a PCR premix (Bioneer, Daejeon, Korea). The internal transcribed spacer (ITS) region was amplified using primers ITS1F and ITS4B (Gardes & Bruns, 1993) under the following conditions: 95 °C for 5 min, 35 cycles of 95 °C for 40 s, 55 °C for 40 s, and 72 °C for 1 min, followed by 72 °C for 5 min. The nuclear large subunit ribosomal RNA (nrLSU) region was amplified with primers LR0R and LR7 (Hopple & Vilgalys, 1994) under the same PCR conditions as those for ITS. The PCR products were electrophoresed on 1% agarose gel to verify the results. They were then purified using the ExpinTM PCR Purification Kit (GeneAll Biotechnology, Seoul, Korea) following the manufacturer’s instructions. Sequencing was performed at Macrogen (Seoul, Korea) with the PCR primers using an ABI Prism 3700 Genetic Analyzer (Life Technologies, Gaithersburg, MD, USA). All ITS and nrLSU sequences were proofread and edited using MEGA X (Kumar et al., 2018).

### Sequence analyses

All ITS and nrLSU sequences belonging to Schizoporaceae were downloaded from the National Center for Biotechnology Information (NCBI) to include sequences described by their former names, such as *Hyphodontia* and *Schizopora*. *Hyphodontia* has been transferred to Hyphodontiaceae (Wang et al., 2021), but the sequences in NCBI were still classified under Schizoporaceae at the time of analysis. Subsequently, sequences from the Basic Local Alignment Search Tool (BLAST) results that grouped phylogenetically with the validated sequences from the present study were analyzed to rectify the misidentifications in the public database. The geographical location for each GenBank sequence was also analyzed for species identification and differentiation. All sequences were obtained on the 2021–07–21.

For each genetic region (ITS and nrLSU), multiple alignments for the phylogenetic analyses were performed using MAFFT version 7 (Katoh & Standley, 2013) with the default settings. Manual trimming was performed at the ends of the alignments. Combined RAxML (Stamatakis, 2006) of ITS and nrLSU regions was constructed with the CIPRES web portal (Miller, Pfeiffer & Schwartz, 2011) using the GTR+G model with 1,000 bootstrap replicates for the maximum likelihood phylogenetic analyses. The sequences used in the present study are listed in Table 2. Representative sequences for each species were deposited in GenBank with accession numbers MZ520578–MZ520585 for ITS sequences and MZ520587–MZ520597 for nrLSU sequences (Table 2).

**Table 2:** Accession numbers used in the present study.

Species	Strain	GenBank accession		Reference
		ITS	nrLSU	
*Xylodon asperus*	2004b	DQ873606	DQ873607	Larsson et al., 2006
	SFC20170209-01	**MZ520578**	**MZ520587**	This study
	SFC20170209-11	**MZ520579**	**MZ520588**	This study
*X. astrocystidiatus*	TNM F24764	NR_154054*	NG_068732*	Yurchenko & Wu, 2014
*X. australis*	CANB569567	MT158703	MT158739	Fernández-López et al., 2020
*X. borealis*	Spirin 9416	MH317760	MH638259	Viner et al., 2018
*X. cystidiatus*	FR-0249200	MH880195	MH884896	Riebesehl et al., 2019
*X. filicinus*	MSK F 12869	NR_163313*	NG_067836*	Riebesehl et al., 2019
*X. flaviporus*	SFC20150211-16	**MZ520581**	**MZ520590**	This study
	SFC20170316-25	**MZ520582**	**MZ520591**	This study
	SFC20180710-24	MK992840	**MZ520592**	Lupala et al., 2019
*X. follis*	FR-0249814	MH880204	MH884902	Riebesehl et al., 2019
*X. hyphodontinus*	KAS-GEL9222	MH880205	MH884903	Riebesehl et al., 2019
*X. kunmingensis*	MSK-F 7381	MH880196	MH884897	Riebesehl et al., 2019
	SFC20170317-07	**MZ520580**	**MZ520589**	This study
	TUB FO 42565	NR_163312*	MH884898*	Riebesehl et al., 2019
*X. magallanesii*	MA:Fungi:90391	MT158720	MT158756	Fernández-López et al., 2020
*X. nespori*	GEL3158	DQ340310	DQ340346	Unpublished
	GEL3290	DQ340309	DQ340343	Unpublished
	SFC20150523-08	**MZ520583**	**MZ520593**	This study
*X. niemelaei*	LWZ20171015-12	MT319625	MT319361	Wang et al., 2021
	KUC20160721B-26	MF774798	**MZ520595**	This study
	GC 1512-1	KX857808	KX857813	Chen, Wu & Chen, 2017
*X. nothofagi*	ICMP 13839	AF145582	MH260064	Unpublished
*X. ovisporus*	FR-0249797	MH880201	MH884901	Riebesehl et al., 2019
	KUC20130808-17	KJ668462	KJ668314	Unpublished
	MA:Fungi:79440	MH260071	MH260066	Unpublished
	SFC20170718-08	**MZ520584**	**MZ520594**	This study
*X. paradoxus*	MA-Fungi_70444	MH260070	MH260065	Unpublished
*X. pseudolanatus*	CFMR FP-150922	NR_163314*	NG_067837*	Riebesehl et al., 2019
*X. quercinus*	MA:Fungi:27435	MT158718	MT158754	Fernández-López et al., 2020
*X. raduloides*	KAS-JR26	MH880225	MH884910	Riebesehl et al., 2019
	MAF 75310	KY962825	KY962864	Fernández-López et al., 2018
*X. serpentiformis*	KUC20121019-31	KJ668517	KJ668369	Unpublished
	TUB-FO 42688	MH880229	MH884913	Riebesehl et al., 2019
*X. spathulatus*	SFC20180818-36	MK992854	**MZ520596**	Lupala et al., 2019
	Wu 1407-105	KX857804*	KX857811*	Chen, Wu & Chen, 2017
	MSK-F 12931	MH880231	MH884914	Riebesehl et al., 2019
*X. subflaviporus*	SFC20180818-15	**MZ520585**	**MZ520597**	This study
	Wu 0809-76	KX857803*	KX857815*	Chen, Wu & Chen, 2017
*X. taiwanianus*	CBS 125875	MH864080	MH875537	Vu et al., 2019
*Hyphodontia pallidula*	GEL2097	DQ340317	DQ340372	Unpublished

**Note:**

Asterisks indicate type sequences, and bolded sequences are those newly generated in the present study.

## Results

### Morphological and molecular identification of *Xylodon* species in Korea

**Figure 1:**
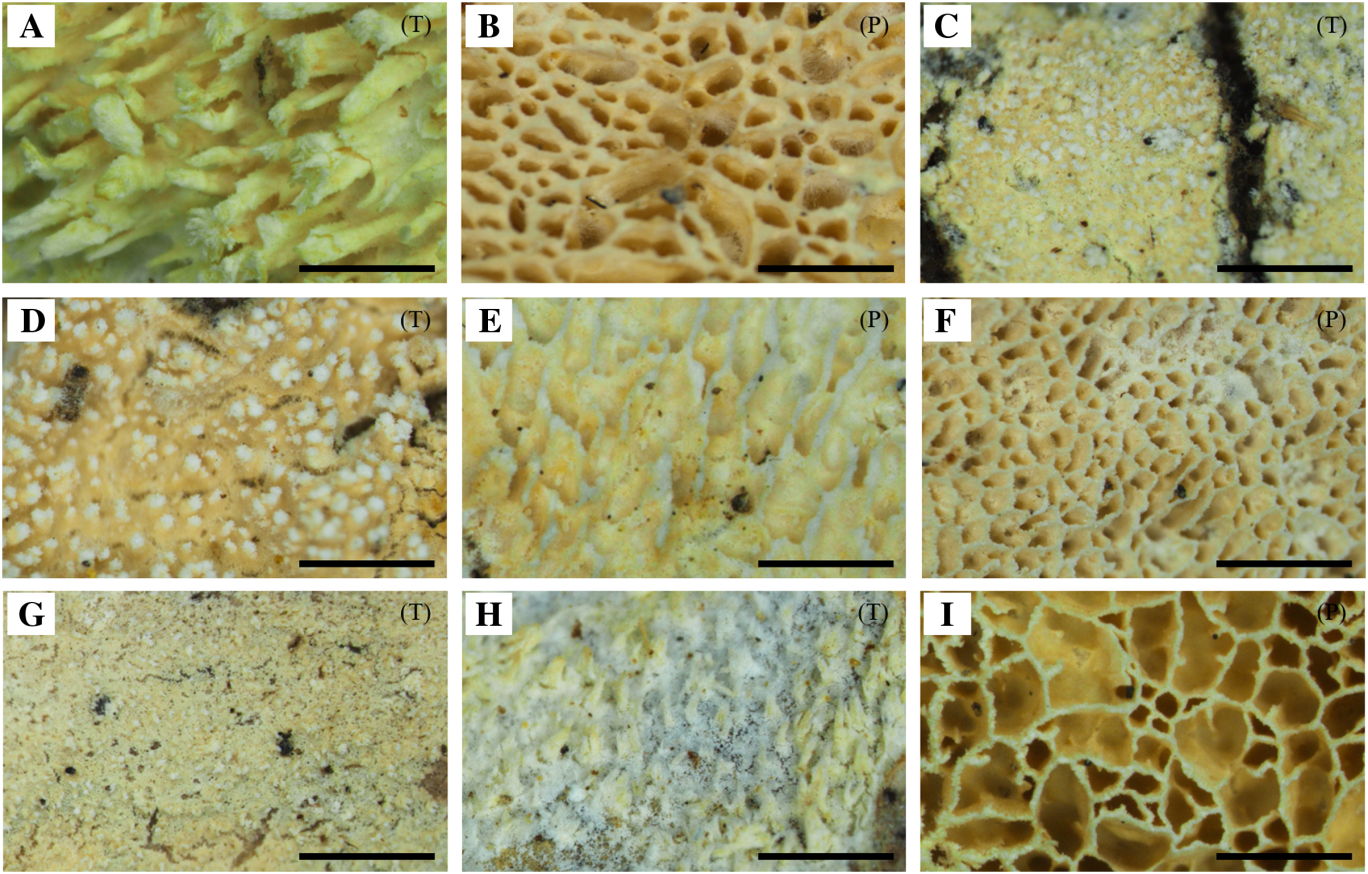
Basidiomes of *Xylodon* species in Korea. (A) *X. asperus* (SFC20170209-11). (B) *X. flaviporus* (SFC20180410-17). (C) *X. kunmingensis* (SFC20160114-24). (D) *X. nespori* (SFC20150523-08). (E) *X. niemelaei* (KUC20160721B-26). (F) *X. ovisporus* (SFC2010718-08). (G) *X. serpentiformis* (KUC20121019-31). (H) *X. spathulatus* (SFC20180818-36). (I) *X. subflaviporus* (SFC20170426-14). Scale bars are 1 mm. ‘(P)’ refers to poroid type and ‘(T)’ refers to toothed type.

**Figure 2:**
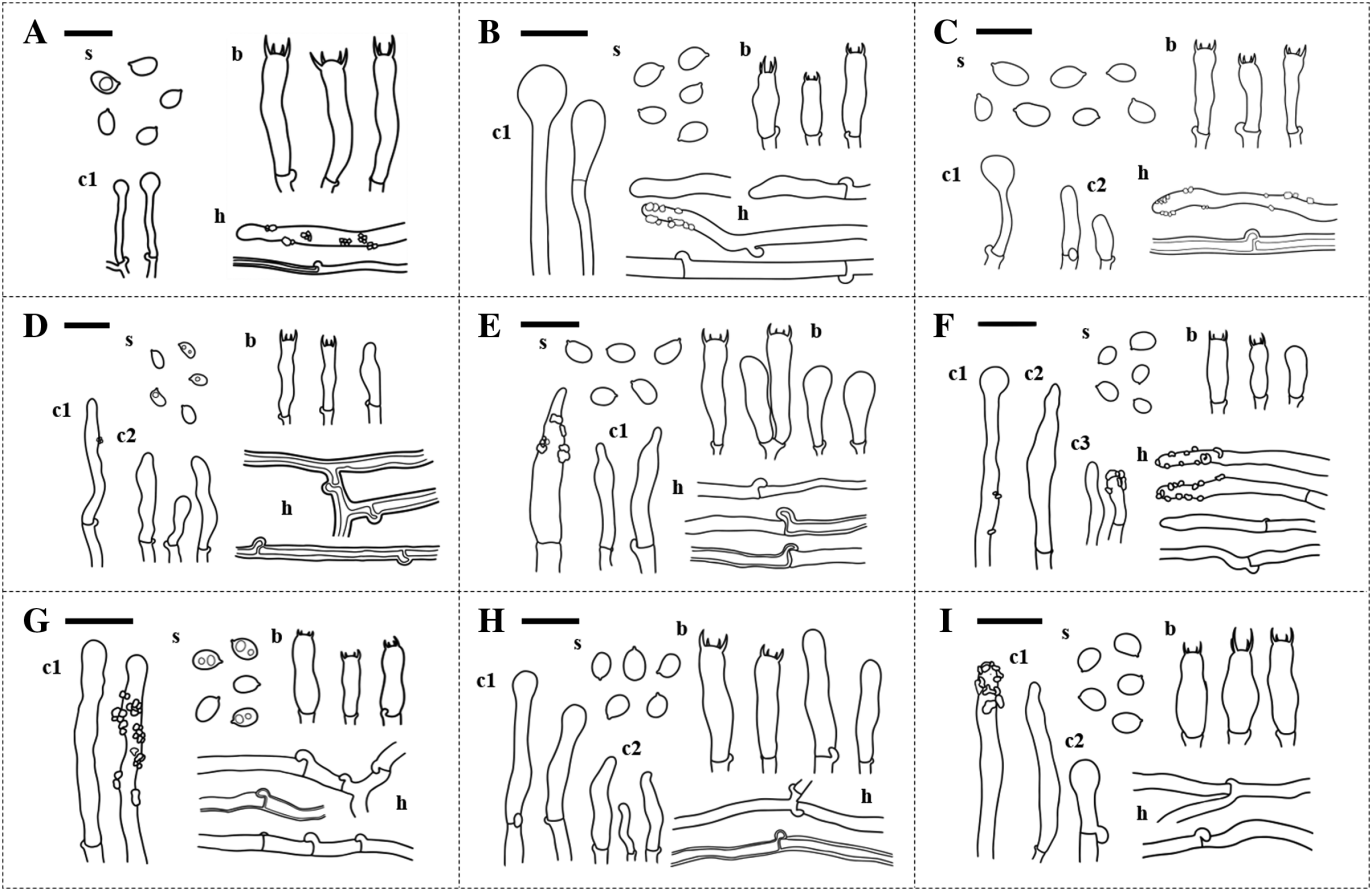
Microscopic characteristics of *Xylodon* species in Korea. (A) *X. asperus*. (B) *X. flaviporus*. (C) *X. kunmingensis*. (D) *X. nespori*. (E) *X. niemelaei*. (F) *X. ovisporus*. (G) *X. serpentiformis*. (H) *X. spathulatus*. (I) *X. subflaviporus*. Scale bars are 10 µm. ‘s’ indicates basidiospores, ‘b’ indicates basidia, ‘c1,’ ‘c2,’ and ‘c3,’ indicate different types of cystidia, and ‘h’ indicates hyphae.

The specimens were divided into nine different groups according to morphology. Features including hymenophore types, pore densities, and basidiospore sizes of *Xylodon* specimens from Korea were analyzed. The basidiomes of all species were resupinate and adnate on wood. There were two hymenophore shapes, raduloid (toothed) and poroid (Fig. 1). Five groups had raduloid hymenophores, and the other four groups were poroid. Through the detailed morphological features, two raduloid groups were identified as *X. asperus* and *X. spathulatus*, and two poroid groups were identified as *X. niemelaei* and *X. ovisporus*. The unidentified raduloid and poroid groups of species were comparably similar in macromorphology, but were divided by several micromorphological characteristics (Table 3; Fig. 2). A taxonomic key to each species is provided below, and detailed morphological characteristics are provided in Table 3. Along with morphological characteristics, the preferential host types of specimens from Korea were compared—host preference of the nine *Xylodon* species was divided into either angiosperms, gymnosperms, or both, and each species corresponded to the respective reference (Tables 1 and 4). All poroid groups and a raduloid species, *X. spathulatus*, had no host preference. The remaining two raduloid groups favored angiosperms, and the other two, including *X. asperus*, preferred gymnosperms.

**Table 3:** Morphological characteristics of *Xylodon* species in Korea.

Characteristics		*X. asperus* ^a^	*X. flaviporus* ^b^	*X. kunmingensis* ^c^	*X. nespori* ^d^	*X. niemelaei* ^e^	*X. ovisporus* ^b^	*X. serpentiformis* ^f^	*X. spathulatus* ^d^	*X. subflaviporus* ^g^
Hymenophore type		raduloid	poroid	odontioid	grandinioid	poroid and arachnoid	poroid	odontioid	raduloid	poroid
Hymenophore color		cream to buff	cream to buff or pinkish buff	cream	cream	cream to buff	cream to buff or pinkish buff	cream	cream	cream to buff
Hyphal system		monomitic	pseudodimitic	monomitic	monomitic	monomitic	pseudodimitic	pseudodimitic	monomitic	pseudodimitic
Clamp connections		present	present	present	present	present	present	present	present	present
Cystidia		capitate, subulate	acicular, apically-encrusted, capitate	capitate	encrusted, subcapitate	capitate, short	acicular, apically-encrusted, capitate	encrusted, tubular	capitate, cylindrical, subulate	acicular, apically-encrusted, capitate
Basidia	shape	suburniform	suburniform	utriform	subclavate to suburniform	subclavate to suburniform	suburniform	suburniform	subclavate to suburniform	suburniform
	length/µm	9.7–18.8	9.7–18.8	**15.0–22.0** **(23.0–27.0)**	15.0–20.0	**13.0–18.0** **(17.0–23.0)**	8.0–15.6	**11.3–14.7** **(15.0–17.0)**	14.0–21.0	11.3–15.4
	width/µm	3.5–5.6	3.5–5.6	3.0–5.3	3.6–4.1	4.0–5.0	3.1–5.1	**2.8–3.9** **(4.0–5.0)**	3.5–5.0	**4.3–6.4** **(4.0–5.0)**
Basidio-spores	ornamentation	ellipsoid	ellipsoid	narrowly ellipsoid	narrowly ellipsoid	ellipsoid	ellipsoid	ellipsoid	ellipsoid	ellipsoid
	length (l)/µm	4.2–5.2	4.2–5.2	5.0–6.3	**4.4–5.1** **(5.0–6.0)**	5.0–6.2	3.5–4.4	4.8–5.8	4.8–5.8	3.9–4.8
	width (w)/µm	3.0–4.0	3.0–4.0	2.5–3.4	2.1–2.6	3.2–3.7	2.6–3.3	3.3–4.3	3.5–4.5	2.8–3.5
	mean (l × w)	4.6 × 3.2	4.6 × 3.2	5.7 × 3.2	4.6 × 2.4	4.6 × 3.5	3.9 × 2.9	5.3 × 3.8	5.3 × 3.9	4.4 × 3.2
	Q value	**1.4** **(1.2–1.3)**	1.4	1.8	1.9	1.6	1.3	1.4	1.4	1.4

**Notes:**

Measurements in bold are deviant from the references, which are given in parentheses.

a Kotiranta & Saarenoksa, 2000.

b Riebesehl & Langer, 2017.

c Shi et al., 2019.

d Eriksson & Ryvarden, 1976.

e Wu, 1990.

f Langer et al., 1992.

g Chen, Wu & Chen, 2018.

**Table 4:** Hymenophores, host types, and geographical distributions of the *Xylodon* species in Korea. The species are ordered phylogenetically. Preferred host types are indicated by asterisks, and geographical distributions are indicated by colored bars.

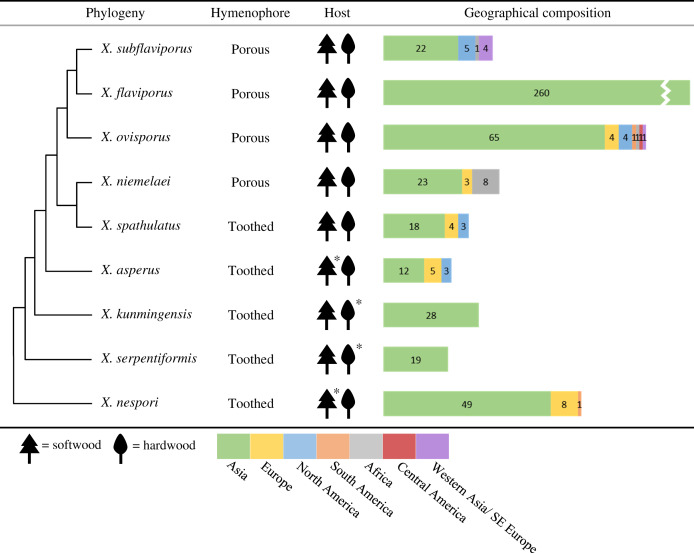

**Figure 3:**
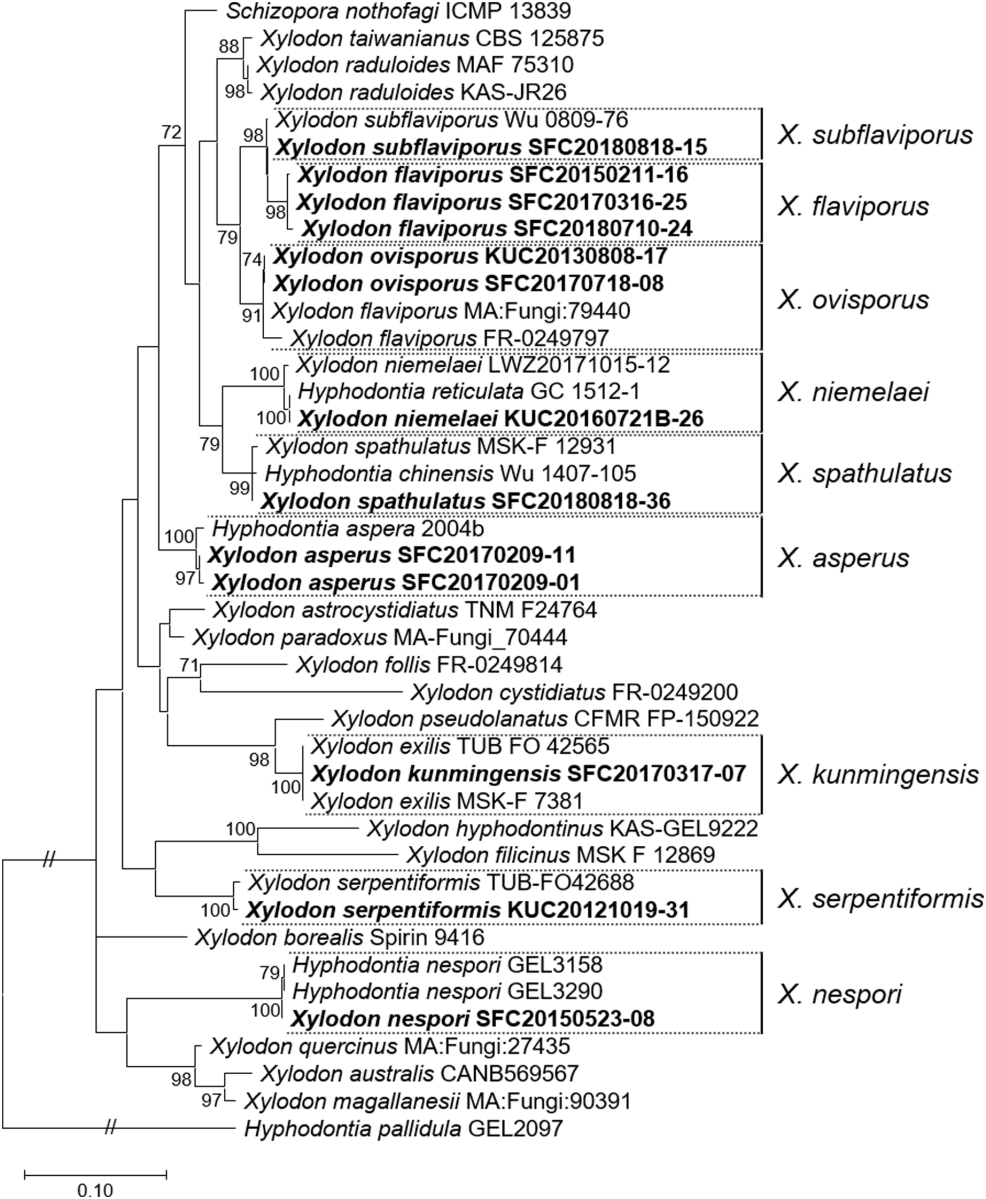
Maximum likelihood (ML) tree of *Xylodon* species constructed based on ITS and nrLSU regions. *Hyphodontia pallidula* (DQ340317) was used as an outgroup. Sequences from Korea are indicated in bold. Bootstrap values >70 are shown.

Five to ten representative specimens from each morphological group were selected for sequencing to verify their identities and distinguish enigmatic species. Nine different taxa were confirmed through the analyses of ITS and nrLSU, corresponding to the morphological groupings: five raduoid species (*X. asperus*, *X. kunmingensis*, *X. nespori*, *X. serpentiformis*, and *X. spathulatus*) and four poroid species (*X. flaviporus*, *X. niemelaei*, *X. ovisporus*, and *X. subflaviporus*). In the public database, type sequences for ITS were available for three of the nine species: *X. kunmingensis* (MK404532, and NR_163312 as *X. exilis*), *X. spathulatus* (as *X. bubalinus*, NR_154097), and *X. subflaviporus* (NG_068781); they were included in the phylogenetic analyses to obtain a more reliable conclusion (Fig. 3).

The concatenated sequences for the maximum likelihood (ML) tree consisted of 578 bases of the ITS region and 517 bases of the nrLSU region. No reference sequence was available for *X. flaviporus* in the nrLSU region from other countries. *Xylodon nespori*, *X. ovisporus*, *X. serpentiformis*, and *X. subflaviporus* sequences from Korea were marginally different from those of the other countries. For instance, *X. subflaviporus* (SFC20180818-15) was different from the holotype (Wu 0809-76) only in two separate regions, one base each in the ITS and nrLSU regions. Neighbor-joining (NJ) tree of ITS for *X. asperus* was constructed to present the division of clades by the geographical origin, and for *X. niemelaei* and *X. spathulatus* to display the related species names that are parallel or highly similar in sequence (Fig. S1).

### Sequence validation

A total of 646 GenBank ITS and nrLSU sequences corresponded to the nine *Xylodon* species in Korea. For ITS, there were 553 sequences with 404 (73.1%) labeled with misleading names and 93 nrLSU sequences with 57 (61.3%) sequences labeled with misleading names (Tables S1 and S2). The misleading names included misidentified, synonymous names, and labels such as “*Hyphodontia* sp.,” “*Xylodon* sp.,” “Fungal sp.,” and “Uncultured fungus”.

*Xylodon flaviporus*, *X. ovisporus*, and *X. subflaviporus* had ITS sequences misidentified as one another, along with sequences identified as *Hyphodontia tropica* (Table S1). *Xylodon flaviporus* had the most ITS sequences (*n* = 260) in GenBank, but only nine were rightly annotated. The remaining 251 sequences were misidentified as *X. ovisporus* (*n* = 63) and *H. tropica* (*n* = 188). *Xylodon ovisporus* had the second largest number of ITS sequences (*n* = 77) in GenBank, with nine annotated correctly. The rest of the sequences were either misidentified as *X. flaviporus* (*n* = 63) under genera *Hyphodontia*, *Schizopora*, and *Xylodon* or as unspecified (*n* = 5). Of the 58 ITS sequences defined as *X. nespori*, 54 were correctly labeled, one was mislabeled as *X. magallanesii*, and three were ambiguously labeled (‘uncultured *Hyphodontia’* and ‘uncultured fungi’). Most ITS sequences of *X. asperus* and *X. kunmingensis* were correctly annotated as to their current name or synonyms. For *X. niemelaei*, 19 ITS sequences were assigned as *X. niemelaei* or *H. niemelaei*. Three other *Xylodon* species names showed high sequence similarity with *X. niemelaei*. Similarly, ITS sequences defined as *X. spathulatus* included *X. bubalinus*, *X. chinensis*, and ambiguously labeled sequences (‘*Xylodon* sp.’ and ‘*Hyphodontia* sp.’). Only for *X. serpentiformis*, all GenBank references were labeled accurately, except for the query ITS sequence from Korea (KJ668517), which was annotated as ‘*Hyphodontia* sp. 1’.

For nrLSU, *X. kunmingensis* and *X. serpentiformis* had no mislabeled sequences, and sequences for the remaining species were revised for their identity (Table S2). *Xylodon asperus* and *X. nespori* had sequences labeled as *Hyphodontia* species. The nrLSU sequences of *X. flaviporus* and *X. ovisporus* had sequences misidentified as one another, similar to the trend seen for ITS. The complexity of *X. niemelaei* and *X. spathulatus* was also reflected in the nrLSU sequences.

For all nine *Xylodon* species, public ITS sequences from Asia were present (Table 4); and three of the nine species, *X. flaviporus*, *X. kunmingensis*, and *X. serpentiformis*, had sequences only from Asia. The other six species were from various parts of the world. *Xylodon ovisporus* sequences were reported from most regions, including Africa, the Americas, Asia, and Europe. *Xylodon asperus* and *X. spathulatus* sequences were from Asia, Europe, and North America. *Xylodon nespori* sequences were from Asia, Europe, and South America. *Xylodon niemelaei* sequences were from Africa, Asia, and Europe. *Xylodon subflaviporus* sequences were from Africa, Asia, and North America. For sequences that had no description of the country of origin, it was presumed that they belonged to the country where the authors or depositors were affiliated.

### Taxonomic key to *Xylodon* in Korea

1 Hymenophore raduloid 2
1* Hymenophore poroid 6
2 Aculei length up to 2 mm *X. asperus*

2* Aculei length <1 mm 3
3 Aculei length up to 0.8 mm; 1–2 aculei per mm *X. spathulatus*

3* >10 aculei per mm 4
4 Up to 25 aculei per mm *X. serpentiformis*

4* 10< aculei <20 per mm 5
5 Hymenophore cream to sand; aculei length up to 200 µm long; up to 12 aculei per mm; basidiospores 4.4–5.1 × 2.1–2.6 µm *X. nespori*

5* Hymenophore cream to buff; aculei length <200 µm; up to 15 aculei per mm; distorted capitate cystidia; bigger basidiospores of 5.0–6.3 × 2.5–3.4 µm *X. kunmingensis*

6 Hymenophore pores arachnoid *X. niemelaei*

6* Hymenophore pores not arachnoid 7
7 >4 pores per mm *X. ovisporus*

7* <4 pores per mm 8
8 Hymenophore cream to buff or pinkish buff; basidiospores 4.2–5.2 × 3.0–4.0 µm *X. flaviporus*

8* Hymenophore cream to buff; more lacerated pore dissepiments; smaller basidiospores of 3.9–4.8 × 2.8–3.5 µm *X. subflaviporus*

## Discussion

### Update on the taxonomy of *Xylodon* in Korea

Through the integration of recent taxonomic revisions of the genus *Xylodon*, nine species were confirmed to occur in Korea. Three *Xylodon* species were reported new to the country: *X. kunmingensis*, *X. serpentiformis*, and *X. subflaviporus*. The remaining six species have previously been reported to reside in Korea, some as *Hyphodontia* or *Schizopora*. For example, *X. asperus* has been reported as *H. aspera*, as a synonym of *H. granulosa* (Lee & Jung, 2005), and *X. niemelaei* has been reported by its synonym, *H. reticulata* (Kwon et al., 2018).

Most of the *Xylodon* specimens collected in Korea were *X. flaviporus*, *X. ovisporus*, and *X. subflaviporus*. They were also abundant nationwide. The lack of clear distinction between these species brought confusion in Korea for a long time. In terms of morphological characteristics, basidiospore dimensions vary among these three species (Table 3; Riebesehl & Langer, 2017; Chen, Wu & Chen, 2018). Their pore sizes are also comparable, with *X. ovisporus* specimens having relatively dense and smaller pores (>4 pores per mm) than those of *X. flaviporus* and *X. subflaviporus* (<4 pores per mm). Furthermore, the pores of *X. subflaviporus* were found to be uneven in shape, with more lacerated pore dissepiments. In Korea, many *X. flaviporus*, *X. ovisporus*, and *X. subflaviporus* specimens have been recorded as *Schizopora paradoxa* (now *Xylodon paradoxus*; Lee et al., 1992; Lim & Jung, 2001). However, our study showed that *X. paradoxus* does not reside in Korea, which was consistent with the results in Fernández-López et al. (2018), where *X. paradoxus* was phylogenetically proven to reside only in Europe.

Both *Xylodon exilis* and *X. kunmingensis* have been reported as new to science in 2019 but were recognized as conspecific, with *X. kunmingensis* having priority (Wang et al., 2021). Wang et al. (2021) have also assessed the monophyletic clade of *X. niemelaei* with its neighboring species, *X. apacheriensis*, *X. reticulatus*, and *X. rhizomorphus*, which was also supported in the present study (Fig. S1A). Similarly, *X. spathulatus* has been recognized to be conspecific to *X. bubalinus* and *X. chinensis* (Riebesehl et al., 2019). *Xylodon spathulatus* specimens from Korea have previously been reported as *X. chinensis* based on NCBI BLAST results (Lupala et al., 2019), but after a thorough examination of the specimens, we recognized that they correspond to the descriptions of *X. spathulatus* (Eriksson & Ryvarden, 1976), and support the morphological (Table 3) and phylogenetic (Fig. 3; Fig. S1B) synonymy with *X. bubalinus* (Wang & Chen, 2017) and *X. chinensis* (Chen, Wu & Chen, 2017).

The morphological characteristics of nine *Xylodon* species in Korea corresponded to the descriptions of their respective references (Table 3). However, measurements of basidia and basidiospores of some species were slightly different. The basidia of the specimens from Korea were generally smaller than those from other regions: *Xylodon kunmingensis* from South China (Shi et al., 2019), *X. niemelaei* from Taiwan (Wu, 1990), *X. serpentiformis* from Taiwan (Langer et al., 1992), and *X. spathulatus* from the Northern Europe (Eriksson & Ryvarden, 1976; Table 4); only the basidia of *X. subflaviporus* specimens in Korea were broader than those of the reference specimens from China (Chen, Wu & Chen, 2018). For basidiospores, the basidiospore length of *X. nespori* was shorter than that of the reference from Northern Europe (Eriksson & Ryvarden, 1976). The basidiospores of *X. asperus* from Korea were broader than that of *Hyphodontia aspera* (= *X. asperus*) from Finland (Kotiranta & Saarenoksa, 2000). These micromorphological variations between specimens may result from the geographical distance or the difference in landscape or habitat. More care is required when studying wood-degrading resupinate fungi such as *Xylodon*, as morphological discrepancies are often only observed at a finer scale. The variations were also reflected in the phylogeny. *Xylodon asperus* sequences from Korea were grouped in a clade with most sequences from Asia (Fig. S1C).

The number of sequences for each species was variable worldwide (Table 4), indicating an unevenness of species dispersal or biases of the locations where research was actively conducted. The ITS sequences of *X. flaviporus* were only submitted from Asia, but *X. flaviporus* has been reported to reside in Europe and South America through morphological analyses (Cooke, 1886; Keizer, 1990; Drechsler-Santos, Groposo & Loguercio-Leite, 2008). Similarly, all ITS sequences of *X. serpentiformis* were from Asia, but nrLSU sequence for the same species was available from Germany (AJ406465). As there is such inconsistency in specimen analyses, sequence-based information should not be solely relied upon when identifying or differentiating species.

White-rotting fungi have different mechanisms to degrade hardwood, which is more intractable than softwood (MacDonald & Master, 2012; Couturier et al., 2015). The difference in mechanisms may contribute to the division of species by host type. Most *Xylodon* species in Korea have been found to grow on dead or decayed wood of both angiosperms and gymnosperms (Eriksson & Ryvarden, 1976; Kotiranta & Saarenoksa, 2000; Chen, Wu & Chen, 2017, 2018). Most angiosperm hosts were oak trees (*Quercus* spp., Fagaceae), and most gymnosperm hosts were pine trees (*Pinus* spp., Pinaceae). Some species have been noted to have a preferential host type. *Xylodon kunmingensis* and *X. serpentiformis* were noted to grow more frequently on deciduous woods (Langer et al., 1992), which was also reflected in our study (Table 1). *Xylodon asperus* and *X. nespori* have been reported to mostly grow on conifers (Eriksson & Ryvarden, 1976; Kotiranta & Saarenoksa, 2000). This was also found in our study despite the small number of specimens collected, where the two species were found to grow on red pine (*Pinus densiflora* Siebold & Zucc.). *Xylodon nespori* has been reported in Korea as a fungus that co-occurs with the ant species *Pristomyrmex punctatus* (Lupala et al., 2019). Wood-decay fungi take several measures to disperse basidiospores, including the use of insect vectors. The host preference of insects increases the chance of wood-decaying fungal basidiospore transfer to suitable host types (Stenlid & Gustafsson, 2001). Therefore, interpretation of the preferential host type of each *Xylodon* species could be used to understand the interactions of *Xylodon* species with other organisms.

### Validity of *Xylodon* sequences in GenBank

Public databases are increasingly being used in multifarious ways by researchers from various biological fields (Duck et al., 2016). Researchers have raised concerns about the use of misidentified sequences in succeeding studies (Stavrou et al., 2018; Fort et al., 2021). A substantial number of *Xylodon* ITS and nrLSU sequences were misidentified or not updated in taxonomy in GenBank (Tables S1 and S2). There were more ITS sequences available in GenBank than nrLSU, possibly owing to the ITS region being a universal DNA barcode marker for fungi. This resulted in more ITS sequences labeled with misleading names. Initial misperceptions of sequences led to an accumulation of mislabeled sequences that were deposited based on identity designated through a BLAST search. For *Xylodon* species, many sequences were still labeled as *Hyphodontia* spp., even after the transfer of some species from *Hyphodontia* to *Xylodon* (Hjortstam & Ryvarden, 2009; Riebesehl & Langer, 2017).

In GenBank, *Xylodon flaviporus* and *X. subflaviporus* had many ITS sequences labeled as *Hyphodontia tropica* (Table S1). We recognized the *H. tropica* sequences as part of the genus *Xylodon*, as of Yurchenko & Wu (2016), who integrated *H. tropica* (nom. inval.) into *X. ovisporus*. However, our study re-identified most of the *H. tropica* sequences as *X. flaviporus*. Thorough evaluation of the morphology and phylogeny of *X. flaviporus* and *X. ovisporus* specimens in the present study revealed that many GenBank ITS sequences of *X. flaviporus* and *X. ovisporus* were identified as one another (Table S1). The two species in this study were identified based on the morphological characteristics (Table 3), which agreed with previous descriptions (Wu, 2001; Yurchenko & Wu, 2016; Riebesehl & Langer, 2017; Chen, Wu & Chen, 2018). However, some of these previous studies did not analyze phylogeny, whereas few studies identified species solely based on sequence alignment at the beginning of GenBank sequence uploads of the two species (Paulus et al., 2000; Buzina et al., 2003). These factors led to misalignments between morphology and sequences of specimens, and this further led to an accumulation of misidentified sequences in GenBank.

Many correctly identified sequences of *Xylodon* species have been described as *Hyphodontia*, or *Schizopora*, possibly due to the shortage of updates upon taxonomic revisions by the authors responsible for sequence uploads. As amendments to GenBank submissions are managed by the submitters (Benson et al., 2004), it is essential for all taxonomists to consistently stay aware of the changes made in the classification and taxonomy of organisms and request essential revisions to NCBI (Schoch et al., 2020). Miscellaneous *Xylodon* sequences that were inaccurate or repetitious in the genus classification exemplify the need for comprehensive research on the taxonomic history of the genus, consistent updates, and morphological observations to prevent the submission and buildup of misleading sequences. The validated sequence information of *Xylodon* species in this study will reduce the errors for sequence-based identification and contribute to the subsequent research based on accurate identification.

## Conclusion

We report nine *Xylodon* species in Korea based on taxonomic descriptions and molecular analyses. Some of the nine species were previously grouped under the genera *Hyphodontia* or *Schizopora*. Through the integration of the upgrade in taxonomy, species that were previously placed in *Hyphodontia* and *Schizopora* were synonymized under the genus *Xylodon*, resulting in nine *Xylodon* species being present in the country today. We provide a taxonomic key and assess the host preference and global distributions of the nine species described here for species delimitation. We also validated the reference sequences on a public database in the hopes of avoiding future misidentification of sequences and preventing further accumulation of mislabeled sequences in GenBank.

## Supplemental Information

10.7717/peerj.12625/supp-1 Supplemental Information 1 Neighbor-joining tree of *Xylodon* species. (A) *X. niemelaei* clade. (B) *X. spathulatus* clade. (C) *X. asperus* clade.

10.7717/peerj.12625/supp-2 Supplemental Information 2 Revised identification of GenBank ITS sequences in accordance with the *Xylodon* ITS sequences generated in this study.

10.7717/peerj.12625/supp-3 Supplemental Information 3 Revised identification of GenBank nrLSU sequences in accordance with the *Xylodon* sequences generated in this study.

## Additional Information and Declarations

### Data Availability

The following information was supplied regarding data availability: The internal transcribed spacer (ITS) and the nuclear large subunit ribosomal RNA (nrLSU) sequences are available at GenBank: MZ520578–MZ520585 for ITS and MZ520587–MZ520597 for nrLSU.
