# Supplementary figures and images for: Taxonomic evaluation of Xylodon (Hymenochaetales, Basidiomycota) in Korea and sequence verification of the corresponding species in GenBank
Source: PeerJ. 2021 Dec 10;9:e12625. doi: 10.7717/peerj.12625 (PMC8667721; doi:10.7717/peerj.12625)

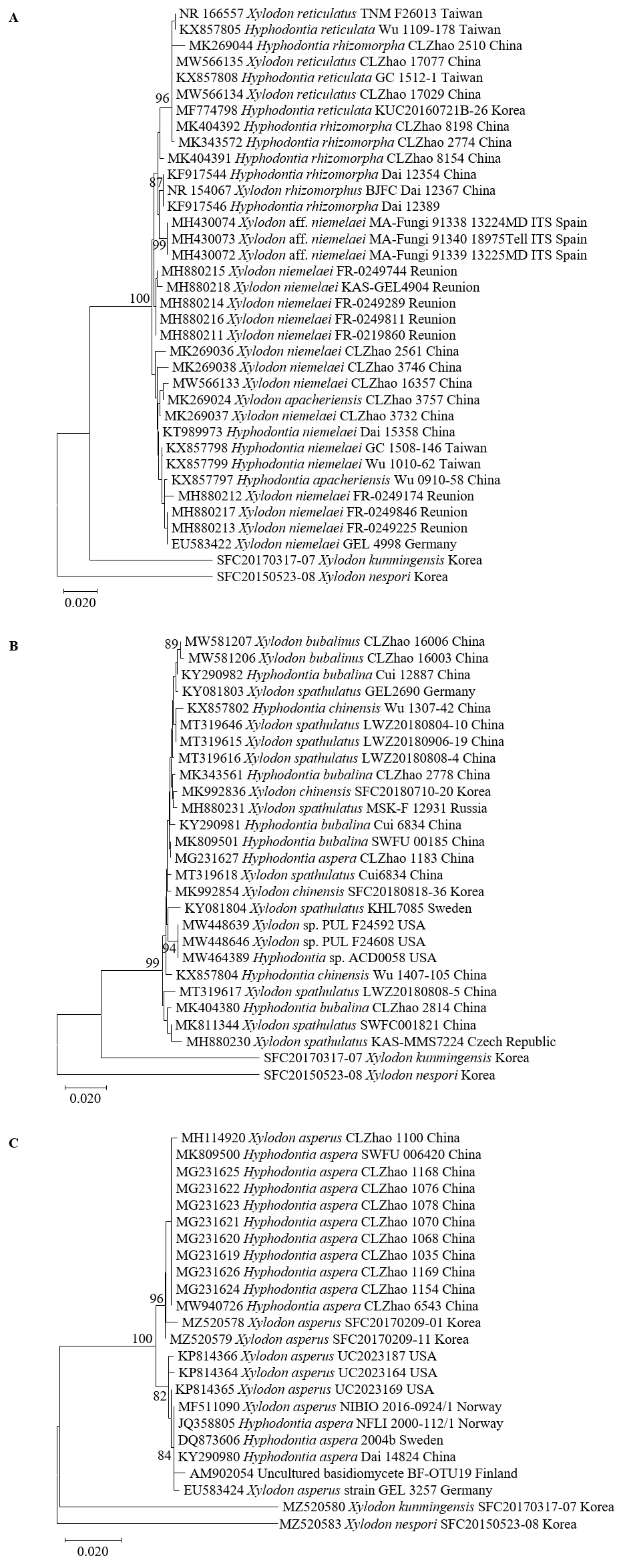

Supplement: Supplemental Information 1 — (A) X. niemelaei clade. (B) X. spathulatus clade. (C) X. asperus clade. [file peerj-09-12625-s001.png]
