# Supplementary material for: Taxonomic evaluation of Xylodon (Hymenochaetales, Basidiomycota) in Korea and sequence verification of the corresponding species in GenBank
Source: PeerJ. 2021 Dec 10;9:e12625. doi: 10.7717/peerj.12625 (PMC8667721; doi:10.7717/peerj.12625)
Supplement: Supplemental Information 2 [file peerj-09-12625-s002.docx]

**Supplemental Table S1. Revised identification of GenBank ITS sequences in accordance with the *Xylodon* ITS sequences generated in this study.**

| Species | Description | Accession Numbers |
| --- | --- | --- |
| *X. asperus* | *Xylodon asperus* | MH114920, KP814365, MF511090, KP814366, KP814364, EU583424 |
|  | *Hyphodontia aspera* | MK809500, MG231626, MG231625, MG231624, MG231623, MG231622, MG231621, MG231620, MG231619, MW940726, KY290980, JQ358805, DQ873606 |
|  | uncultured Basidiomycota | AM902054 |
| *X. flaviporus* | *Xylodon flaviporus* | MF540763, MK343690, MF540761, MK838856, MK809478, MK838853, MK992840, MK838888, MK809470 |
|  | *Xylodon ovisporus* | MK268889, MT319574, MT319608, MT319593, MT319582, MT319595, MT319581, MT319580, MK268890, MT319600, MT319578, MT319663, MT319606, MT319648, MT319579, MT319597, MK269272, MK269015, MT319592, MT319576, MT319596, MT319653, MT319584, MT319652, MT319650, MT319607, MT319603, MT319598, MT319594, MT319585, MT319577, MT319573, MT319572, MK268859, MK268858, MK268841, EU583421, MH114921, MT319601, MT319669, MT319667, MT319666, MT319649, MT319605, MT319604, MT319602, MT319599, MT319589, MT319586, MT319583, MT319575, MT319571, MT319591, MT319588, MT319587, MT319590, MK269312, MK269311, MK269310, MK268882, MK268868, MH114923, MH114922 |
|  | *Hyphodontia tropica* | MK269057, MK343576, KJ668513, MK269054, MH114749, MK269059, MK404398, MK343578, MK811279, MH114753, MW578299, MK404397, MK404396, MK343585, MK343583, MK343582, MK343580, MK269080, MK269076, MK269074, MK269062, MK269051, MK811270, MK811269, MK811267, MK811266, MK811263, MK795157, MK795156, MK795154, MK795153, MK795150, MH114783, MH114779, MH114777, MH114775, MH114771, MH114763, MH114760, MH114751, MH114750, MW742639, MW578305, MW578303, MK811265, MK795152, MK404395, MK343577, MK269069, MK269052, MK811268, MK795155, MH114778, MW742640, MW578298, MK404394, MK343584, MK343581, MK269078, MK269075, MK269073, MK269070, MK269066, MK269061, MK811271, MK795158, MH114776, MW742649, MW742648, MW742647, MW742645, MW578297, MK269077, MK269065, MK269060, MH114761, MK269048, MK269068, MW578302, MG231665, MK269072, MG231679, MG231659, MW578296, MW578362, MH114759, MW742642, MK811272, MK795159, MK269056, MH114781, MH114754, MW742650, MW578304, MW578294, MK269071, MK269064, MK811264, MK795151, MH114787, MH114785, MH114784, MH114782, MH114772, MH114770, MH114752, MG231692, MG231673, MG231669, MG231661, MG231660, MW742651, MW578307, MH114766, MH114762, MK269079, MK811274, MK811273, MW940716, MW940714, MW862306, MW742646, MW742644, MW742643, MW742636, MW742634, MW578306, MG231650, MW742641, MW742633, AF145584, MK269058, MK269055, MK269053, MK269050, MK269049, MH114786, MH114774, MH114773, MH114769, MH114768, MH114765, MH114764, MH114757, MH114756, MG231691, MG231690, MG231689, MG231688, MG231687, MG231686, MG231685, MG231684, MG231683, MG231682, MG231681, MG231680, MG231678, MG231677, MG231676, MG231675, MG231672, MG231667, MG231666, MG231664, MG231662, MG231658, MG231657, MG231656, MG231654, MG231652, MW578301, MW578300, MW578295, MH114780, MG231674, MG231668, MG231655, MG231653, MG231651, MW940715, MW742638, MW742637, MW742635, MW578293, MW578292, MW578291, MG231693 |
| *X. kunmingensis* | *Xylodon kunmingensis* | MW566115, MW566116, MW566117, MW566118, MW566119, MW566120, MW566121, MW566122, MW566123, MW566124, MW566125, MW566126, MW566127, MW566128, MW566129, MK404528, MK404531, MK404532, MK404529, MK404530 |
|  | *Xylodon exilis* | MH880196, MH880197, MH880198 |
|  | *Stereum sanguinolentum* | MK269294, MK269299, MK269308, MK404506, MG231848 |
| *X. nespori* | *Xylodon nespori* | MH880210, MH114743, MH114742, MG231643, MT319675, MT319647, MW940733, DQ873622, DQ340309, DQ340307, MK269035, MK809407, MK795196, MH114741, MH114739, MH114738, MH114737, MH114736, MG231645, MG231644, MG231642, MG231641, MG231640, MG231639, MG231638, MG231637, MG231635, MW566130, DQ340308, MF774797, MK404381, MK343570, MK894107, MK809408, MG231646, MW940734, MT319495, MK343569, MK809409, MK992823, MW566131, MT319662, MT319661, MK992855, KJ668518, MH114740, MT319494, MT319493, MT319655, MT319492, MT319491, MT319490, MT319489, MT319488 |
|  | *Xylodon magallanesii* | MT158721 |
|  | uncultured *Hyphodontia* | KF639810 |
|  | uncultured fungus | FJ820644, MK796531 |
| *X. niemelaei* | *Xylodon niemelaei* | EU583422, MW566133, MK269036, MK269037, MK269038, MH880211, MH880212, MH880213, MH880214, MH880215, MH880216, MH880217, MH880218 |
|  | *Xylodon* aff. *niemelaei* | MH430072, MH430073, MH430074 |
|  | *Xylodon apacheriensis* | MK269024 |
|  | *Xylodon reticulatus* | NR_166557, MW566134, MW566135 |
|  | *Xylodon rhizomorphus* | NR_154067 |
|  | *Hyphodontia niemelaei* | KT989973, KX857798, KX857799 |
|  | *Hyphodontia apacheriensis* | KX857797 |
|  | *Hyphodontia reticulata* | MF774798, KX857808, KX857805 |
|  | *Hyphodontia rhizomorpha* | MK269044, MK343572, MK404391, MK404392, KF917544, KF917546 |
|  | *Hyphodontia rhizomorpha* | MK269044, MK343572, MK404391, MK404392, KF917544, KF917546 |
| *X. ovisporus* | *Xylodon ovisporus* | MK404527, MF540764, MK992818, MK992819, MK992846, MK992850, MK992859, MF540766, MF540765 |
|  | *Xylodon flaviporus* | MT571531, MW742632, MH114732, MH114733, MH114734, MH114735, MK269270, MK269026, MH114928, MK269271, MK269027, MK269028, MK269029, MK269030, MH880201, MH880202, MH880203, MH260071, MT044406, AF145585, MK920119, MT319522, MT319523, MT319524, MT319542, MT319543, MT319544, MT319545, MT319546, MT319547, MT319548, MT319549, MT319550, MT319551, MT319552, MT319553, MT319554, MT319555, MT319556, MT319557, MT319558, MT319559, MT319560, MT319561, MT319562, MT319563, MT319564 |
|  | *Hyphodontia flavipora* | MH398544, AF455399, KJ140637, KJ140642, KJ140665, MG231634, MG231630, MG231631, MG231632, MG231633, KP212089, KJ668462 |
|  | *Hyphodontia* sp. | MH267968 |
|  | *Schizopora flavipora* | AF145575, AF145573, AF145574, AF145585 |
|  | Fungal sp. | JQ919951 |
|  | Uncultured fungus | KF800602, JX984688, JX984747 |
| *X. serpentiformis* | *Xylodon serpentiformis* | MT319673, MT319478, MW566141, MW566138, MH880228, MT319668, MW566139, MW566137, MT319477, MT319665, MT319476, MW566136, MH880229, MH880227, MT319672, MT319664, MW566140, MT319475, MW566142 |
| *X. spathulatus* | *Xylodon spathulatus* | MK811344, KY081803, MH880231, KY081804, MT319618, MT319616, MH880230, MT319617, MT319646, MT319615 |
|  | *Xylodon bubalinus* | MK809501, MK404380, KY290981, KY290982, MW581207, MW581206, MK343561 |
|  | *Xylodon chinensis* | MK992854, MK992836, KX857804, KX857802 |
|  | *Xylodon* sp. | MW448639, MW448646 |
|  | *Hyphodontia aspera* | MG231627 |
|  | *Hyphodontia* sp. | MW464389 |
| *X. subflaviporus* | *Xylodon subflaviporus* | MT319570, MT319568, MF540768, MK343691, MK894112, MF540767, MT319569, MT319566, MT319567, MN749657, MH880233, KX857803, MN749656, MK894113, MK894111 |
|  | *Xylodon ovisporus* | MK269313, MK269273 |
|  | *Xylodon* sp. | MT319565 |
|  | *Hyphodontia tropica* | KY449375, MN752433, KC414243, MH114758, MN752432, KY264047, KY264045, MK269067, MH399868, MH114755, KY264046, AF145587 |
|  | Fungal sp. | LC520140 |
|  | Uncultured fungus | KF800611 |
