# Supplementary material for: Taxonomic evaluation of Xylodon (Hymenochaetales, Basidiomycota) in Korea and sequence verification of the corresponding species in GenBank
Source: PeerJ. 2021 Dec 10;9:e12625. doi: 10.7717/peerj.12625 (PMC8667721; doi:10.7717/peerj.12625)
Supplement: Supplemental Information 3 [file peerj-09-12625-s003.docx]

**Supplemental Table S2. Revised identification of GenBank nrLSU sequences in accordance with the *Xylodon* sequences generated in this study.**

| Species | Description | Accession Numbers |
| --- | --- | --- |
| *X. asperus* | *Hyphodontia aspera* | DQ873607 |
| *X. flaviporus* | *Xylodon ovisporus* | MT319327, MT319319, MT319320, MT319321, MT319323, MT319325, MT319326, MT319330, MT319331, MT319332, MT319333, MT319335, MT319336, MT319337, MT319338, MT319339, MT319340, MT319343, MT319346, MT319287, MT319289, MT319312, MT319314, MT319322, MT319316, MH260063 |
| *X. kunmingensis* | *Xylodon kunmingensis* | MT319258, MT319259 |
| *X. nespori* | *Xylodon nespori* | MT158753, MT319237, MT319236, MT319240, MT319239, MT319238, MT319234, MT319235 |
|  | *Hyphodontia nespori* | DQ873622, AJ406457 |
|  | *Hyphodontia* aff. *breviseta* | AJ406456 |
| *X. niemelaei* | *Xylodon niemelaei* | MT319358, MT319359, MT319360, MT319361, MT319362, MT319363, MT319364, MT319365 |
|  | *Xylodon* aff*. niemelaei* | MH430071 |
|  | *Hyphodontia niemelaei* | KX857816, KX857817 |
|  | *Hyphodontia reticulata* | KX857813 |
| *X. ovisporus* | *Xylodon ovisporus* | MT319317 |
|  | *Xylodon flaviporus* | MT319292, MT319283, MT319282, MT319281, MT319280, MT319279, MT319278, MT319277, MT319276, MT319274, MT319273, MT319272, MT319271, MT319270, MT319290, MT319275, MH260066, MT319284, MT319291, MH878361 |
|  | *Xylodon* sp. | MT319285 |
| *X. serpentiformis* | *Xylodon serpentiformis* | MH884913, MT319220, MT319219, MT319218, MT319217, MT319216, MT319214, MT319213, MT319212, MH884912, MT319215 |
| *X. spathulatus* | *Xylodon spathulatus* | MT319351, MT319352, MT319353, MT319354 |
|  | *Xylodon chinensis* | KX857810 |
| *X. subflaviporus* | *Xylodon subflaviporus* | NG068781, KX857815 |
|  | Fungal sp. | LC520186 |
